# Localized Surface Plasmon Resonance Biosensing: Current Challenges and Approaches

**DOI:** 10.3390/s150715684

**Published:** 2015-07-02

**Authors:** Sarah Unser, Ian Bruzas, Jie He, Laura Sagle

**Affiliations:** Department of Chemistry, College of Arts and Sciences, University of Cincinnati, 301 West Clifton Court, Cincinnati, OH 45221-0172, USA; E-Mails: unsersa@mail.uc.edu (S.U.); bruzasir@mail.uc.edu (I.B.); hej2@mail.uc.edu (J.H.)

**Keywords:** plasmonic, biosensing, noble metal nanoparticles, point-of-care diagnostics

## Abstract

Localized surface plasmon resonance (LSPR) has emerged as a leader among label-free biosensing techniques in that it offers sensitive, robust, and facile detection. Traditional LSPR-based biosensing utilizes the sensitivity of the plasmon frequency to changes in local index of refraction at the nanoparticle surface. Although surface plasmon resonance technologies are now widely used to measure biomolecular interactions, several challenges remain. In this article, we have categorized these challenges into four categories: improving sensitivity and limit of detection, selectivity in complex biological solutions, sensitive detection of membrane-associated species, and the adaptation of sensing elements for point-of-care diagnostic devices. The first section of this article will involve a conceptual discussion of surface plasmon resonance and the factors affecting changes in optical signal detected. The following sections will discuss applications of LSPR biosensing with an emphasis on recent advances and approaches to overcome the four limitations mentioned above. First, improvements in limit of detection through various amplification strategies will be highlighted. The second section will involve advances to improve selectivity in complex media through self-assembled monolayers, “plasmon ruler” devices involving plasmonic coupling, and shape complementarity on the nanoparticle surface. The following section will describe various LSPR platforms designed for the sensitive detection of membrane-associated species. Finally, recent advances towards multiplexed and microfluidic LSPR-based devices for inexpensive, rapid, point-of-care diagnostics will be discussed.

## 1. Introduction

Noble metal nanoparticles, often composed of gold, silver, copper, and aluminum, have proven useful since the 4th century A.D. with the invention of the Lycurgus Cup. The vibrant colors of the Lycergus Cup were created by mixing small amounts of gold and silver into the glass, thus creating nanoparticles embedded in the glass. These nanoparticles are capable of absorbing and scattering light at very specific regions of the visible spectrum, appearing in vivid color to the eye. The main thing that sets these materials apart from other types of materials is their ability to convert the energy of incoming photons (light) into a collective oscillation of electrons. This produces wavelength-selective absorption and scattering of light with molar extinction coefficients as high as 10^11^ M^−1^·cm^−1^, which is several orders of magnitude higher than typical molar extinction coefficients of organic dye molecules. These molar extinction coefficients can also vary depending on nanoparticle size, thus extinction efficiency factors, defined as the extinction coefficient divided by the cross sectional area of the nanoparticle, are also commonly reported and generally range from 3 to 18 for most plasmonic nanoparticles [1]. In addition to wavelength-selective absorption and scattering, the coherent oscillation of electrons at the nanoparticle surface also produces large electromagnetic field enhancements along with radiative decay. These two phenomena have already been shown to be extremely useful in spectroscopic interrogation and heat-mediated release of attached molecules [2,3]. Today, noble metal nanoparticles have found tremendous use in a large range of topics, such as electro-optical and semiconducting materials [4], catalytic materials [5], drug delivery and biological imaging agents [6], and biosensors [7]. This article focuses mainly on the biosensing applications of noble metal nanoparticles.

The use of noble metal nanoparticles in biosensing has grown enormously in recent years, mainly due to several advantages over other traditional biosensing methods. Since the nanoparticles themselves are colored, detection can often be carried out with the naked eye, yielding rapid, inexpensive, portable detection [8]. In addition, the production and fabrication of nanoparticles, which contain small amounts of material, is often inexpensive. The small nature of the nanoparticles and the diversity of platforms available, both in solution and on a surface, also makes this technique amenable to multiplexed, on-chip devices [9,10]. Finally, the remarkable sensitivity of these materials to biological binding has greatly furthered their use in challenging problems where low limits of detection are required.

However, several challenges still remain which limit the use of noble metal nanoparticles in many biosensing applications. This article will focus on four major challenges facing the field of localized surface plasmon resonance (LSPR) biosensing: limit of detection, selectivity, detection of membrane-associated species, and incorporating LSPR devices into multiplexed platforms. Although the ability to detect large biological molecules using plasmonic nanoparticles is well established, the detection of small molecules remains problematic, since a larger number of these molecules are required to coat the nanoparticle surface. In addition, selectivity in complex biological media, such as blood or urine, remains difficult due to the biofouling of the nanoparticle surface. The detection of membrane-associated species, which are more than half of all drug targets, is also challenging since these proteins often require lipids and detergents to interface with the nanoparticle sensors. Finally, although these miniaturized sensors should prove extremely useful for on-chip devices, little work has been done towards realizing this goal.

This article highlights recent advances in the field to overcome the limitations mentioned above. The first section gives a general background to the physical origin of LSPR and factors that affect the observed LSPR response. The next several sections examine recent advances aimed at improving limit of detection, selectivity, amenability towards membrane protein detection, and incorporation of LSPR devices into multiplexed and microfluidic point-of-care diagnostic platforms. Improvements in limit of detection and selectivity have come about through the diversity of complex nanoparticles as well as plasmonic platforms currently available. Other improvements in fabrication have allowed for combining plasmonic and silicate materials to interface with lipid bilayers for membrane protein detection. Lastly, recent studies have been carried out to interface plasmonic materials with multiplexed devices for inexpensive, portable point-of-care medical diagnostics.

### *General Principles of Localized Surface Plasmon Resonance* 

**Figure 1:**
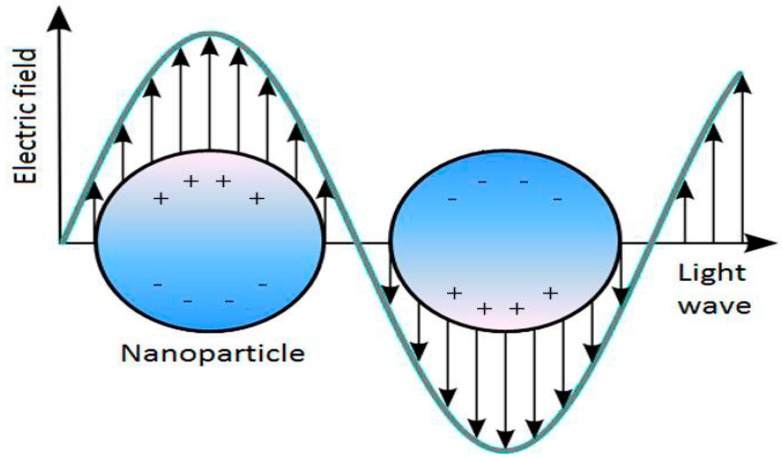
Schematic diagram illustrating the localized surface plasmon on a nanoparticle surface.

The interaction of light with noble metal nanoparticles produces a collective oscillation of conduction band electrons known as the localized surface plasmon resonance (LSPR). Only materials with a negative real and small positive imaginary dielectric constant are capable of supporting surface plasmons. The most common materials used are gold and silver, although other metals such as copper and aluminum also exhibit plasmon resonance. When the incident electromagnetic field matches that of the oscillating electrons on the surface of the nanoparticle, a resonance condition is met [11], see Figure 1.

This resonant oscillation produces large, wavelength-selective increases in absorption, scattering, and electromagnetic field at the nanoparticle surface. The increases in absorption and scattering have been utilized towards LSPR biosensing. The increases in electromagnetic fields have also proven very useful in surface enhanced Raman spectroscopic (SERS) detection of biological analytes. This review will only cover LSPR biosensing and not SERS-based platforms. Please see excellent reviews on SERS-based biosensing elsewhere [12,13,14].

In order to understand the factors that affect the large increase in absorption and scattering when resonance occurs, Mie theory is often evoked [15]. Mie theory is an analytical solution to Maxwell’s equations with spherical boundary conditions, which is used to describe the extinction spectra of a given nanoparticle. In order to more accurately calculate the dielectric constants at the different wavelength values and extend this theory to more complex shapes, the Modified Long Wavelength Approximation (MLWA) of Mie theory is used [16]:

(1) $$\;C_{ext}=\;\frac{24\;{\text{π}}^{2}R^{3}{\text{ε}}_{m}^{\frac{3}{2}}N}{\text{λ}\;ln\left(10\right)}\;\frac{{\text{ε}}_{i}}{{\left({\text{ε}}_{r}+\;{\text{χε}}_{m}\right)}^{2}+\;{\text{ε}}_{i}^{2}\;}$$

where *R* is the radius of the particle, λ is the wavelength of the incident light, ε_m_ is the dielectric constant of the surrounding medium, $\text{ε}=\;{\text{ε}}_{r}+\;i{\text{ε}}_{i}$ is the complex dielectric constant of the bulk metal, *N* is the electron density, and χ accounts for the shape of the particle. This “shape factor”, χ, models the particles as an ellipse and is proportional to a/b where a and b are the minor and major axis respectively of the ellipse. As shown in Equation (1), many factors such as shape of the nanoparticle, wavelength of incident light, type of material, and the surrounding media influence the absorption and scattering processes. In fact, the large effect of the surrounding dielectric constant on the extinction spectra of the plasmonic nanoparticle has been the basis of much of the work carried out in the field of LSPR biosensing.

When a biological analyte binds to the surface of the nanoparticle, a change in refractive index at the nanoparticle surface is induced, which in turn shifts the LSPR peak frequency. This shift of LPSR frequency is also affected by the makeup of the nanoparticle and its shape. Silver, having the largest negative real dielectric constant of all the plasmonic materials, is the most sensitive to changes in local refractive index. In addition, nanoparticles of asymmetric shape are also more sensitive to changes in biomolecular surface binding than spherical colloids. The shift in LSPR frequency upon adsorbate binding has been described by the following relation [17,18]:

(2) $$∆\text{λ}\;=m\;\left(∆n\right)\;\left[1-exp\left(\frac{-2d}{l_{d}}\right)\right]$$

where *m* is the refractive index sensitivity, Δ*n* is the change in refractive index induced by the adsorbate, *d* is the effective adsorbate layer thickness and *l_d_* is the electromagnetic field decay length (approximated as an exponential decay). Thus, two key variables that determine the size of the LSPR shift observed is the difference in refractive index of the absorbate relative to solution (Δ*n*) and the size of the analyte (*d*) that is binding to the nanoparticle surface*.* The refractive index sensitivity (*m*) is often obtained by taking the slope of a plot of LSPR frequency *versus* refractive index, which is predicted to be linear (within a relatively small range of index of refraction) by the Drude model of the electronic structure of metals [19]. Last, the electromagnetic field decay length, *l_d_*, which can also effect the LSPR shift observed, has been shown to be sensitive to the shape of the nanoparticle. This electromagnetic field decay length for many nanoparticles (50–100 nm in diameter) is similar in size to that of a protein molecule, 5–10 nm, which allows for the unique sensitivity of noble metal nanoparticles to sense the binding of biological molecules to the surface [20,21].

Since refractive index sensitivity is key for detecting a biological molecule of interest, many endeavors in recent years have attempted to make nanoparticle substrates that exhibit high sensitivity to changes in refractive index. Interestingly, recent simulations strongly suggest that the refractive index sensitivity increases in a linear fashion as the LSPR frequency of the material shifts from blue to red wavelengths [22,23]. Shifting the LSPR frequency to longer wavelengths can be achieved by creating larger and/or asymmetric structures [24,25]. In addition, when two plasmonic structures come into close contact, their electromagnetic fields couple, which can produce large shifts to longer wavelengths [26,27]. The presence of a nearby plasmonically active species often has a much larger effect on the LSPR frequency than a change in refractive index. Therefore, numerous biosensing studies make use of plasmonic coupling as a means of producing significant changes in detected signal.

In this article, current challenges and approaches in plasmonic biosensing are discussed. The first major challenge addressed is limited sensitivity and detection limits, particularly for refractive-index based biosensing. Although changing the nanoparticle shape and material can improve sensitivity and decrease the limit of detection by as much as 10-fold, amplification techniques can produce a decrease in K_d_ and limit of detection by several orders of magnitude. Thus, the first section will focus on recent amplification techniques to improve the limit of detection. A second major challenge discussed is selectivity, particularly in complex biological fluids. Three major strategies for improving selectivity involve self-assembled monolayers on the nanoparticle surface, the use of biological scaffolds, and size/shape complementarity. Next, the challenge of carrying out biosensing with membrane-associated species through interfacing plasmonic structures with lipid bilayers and vesicles is reviewed. Last, the ultimate goal and challenges associated with incorporating plasmonic devices into multiplexed and microfluidics platforms for simple, facile point-of-care diagnostics are examined.

## 2. The Challenge of Improving Limit of Detection

Although LSPR is a versatile and sensitive technique, it faces challenges that limit its use. Since many LSPR-based biosensing measurements detect changes in refractive index at the nanoparticle surface, the detection of small molecules, which require large numbers to coat the surface, remains problematic. In order to increase the limit of detection of molecules of interest, many strategies that have been implemented. Tuning the nanoparticle material, size and shape may alter the plamonic properties towards heightened sensitivity and decreased limits of detection. Please see reviews on this topic elsewhere [28,29,30,31,32,33,34]. Another prospect is to amplify the signal once the analyte of interest binds to the nanoparticle surface. These amplification strategies are often more effective at producing large measurable changes upon binding small amounts of substrate. The next three Section 2.1, Section 2.2 and Section 2.3, will discuss some common amplification techniques, including enzyme-mediated, plasmonic coupling, and biomolecule conformationally mediated amplification techniques.

### 2.1. Enzymatic Amplification

When a molecule of interest binds to a plasmonic substrate, it is possible to trigger other chemical reactions whose by-products can yield an amplified response. For many biosensing applications, these chemical side-reactions are often carried out using enzymes. One strategy is to use enzymes to induce precipitation of molecules on the surface of the plasmonic structure in response to a biological binding event. An example of enzymatic-mediated precipitation comes from a study by Shin and co-workers, who made nanodisc arrays functionalized with PSA antibody. Upon binding PSA analyte, a second antibody, functionalized with alkaline phosphatase, binds the bound analyte. Alkaline phosphatase then catalyzes the precipitation of 5-bromo-4-chloro-3-indoyl phosphate p-toluidine/nitro blue tetrazolium onto the surface of the gold nanodiscs yielding a larger LSPR shift. This study enabled femtomolar detection of PSA, which is five orders of magnitude lower than without the precipitation reaction [35]. Another method of harnessing precipitation reactions for signal amplification uses the enzyme horseradish peroxidase. Lee *et al.* added horseradish peroxidase to their system to catalyze the precipitation of 4-chloro-1-napthol onto the surface of gold nanoislands to amplify the signal due to binding. As IFN-γ-antigen binds its immobilized receptor, a second biotinylated antibody binds in a traditional sandwich immunoassay scheme. Finally, an avidin protein containing the enzyme horseradish peroxidase binds to the biotinylated antibody, and catalyzes the precipitation of 4-chloro-1-napthol onto the surface yielding detection as low as 0.54 nM [36].

Another enzymatic amplification strategy is to use enzymes that upon substrate binding, produce chemical by-products which can then react directly with the plasmonic substrate. These reactions can often produce large changes in the shape and size of the nanoparticle substrate, which in turn generate increased shifts in LSPR frequency. An example of this approach is demonstrated by a recent study, which used a combination of horseradish peroxidase and glucose oxidase for the detection of glucose. By attaching both enzymes to gold nanorods, the addition of glucose produced H_2_O_2_, which was then used to oxidize and etch the gold nanorods. This etching of the gold nanorods, which was dependent on glucose concentration, produced large blue-shifts in the LSPR spectrum and worked as sensitive, colorimetric glucose detection device in serum [37]. Likewise, other works have used the highly reactive properties of H_2_O_2_ to modify the nanoparticle shape for improved detection schemes. Xia *et al.* used the enzyme glucose oxidase (GOx) mixed in solution with silver nanoprisms to catalyze the reaction between glucose and oxygen to form H_2_O_2_ and gluconic acid. As the reactive H_2_O_2_ etched the tips of the nanoprisms, drastic shape and color changes were observed in the LSPR spectrum [38]. The more glucose present in solution, more H_2_O_2_ is produced, further etching the tips of the nanoprisms which resulted in a detection range from 2.0 × 10^−7^ to 1.0 × 10^−4^ M in diluted serum. The nanoprism etching scheme has also been coupled with GOx and applied to detect fM target DNA [39].

Enzymes have also been used to nucleate reactions directly on nanoparticle surfaces for improved target detection. In order to push the limits of detection of PSA antigen in serum, Rodriguez-Lorenzo *et al.* developed an ultrasensitive assay using gold nanostars [40]. In this assay, the nanostars are functionalized with a primary antibody, which binds small amounts of the PSA antigen. Next, by conjugating the enzyme glucose oxidase to a secondary antibody specific for PSA, a sandwich assay is created on the surface of the gold nanostars. The small amounts of glucose oxidase present on the surface of the gold nanostars were then able to catalyze the growth of a silver shell onto these structures, generating a substantial blue shift for very small amounts of enzyme, and thus PSA present. Interestingly, more complete silver shells were grown for lower PSA concentrations yielding larger plasmonic shifts than higher concentrations of PSA. This unique inverse sensitivity allowed for the lowest possible limit of detection for PSA in whole serum, 4 × 10^−20^ M, to be realized with this approach.

### 2.2. Plasmonic Nanoparticle Coupling-Mediated Amplification

As described in the Introduction, when plasmonic particles come into close proximity to one another, plasmonic coupling can occur, which can lead to drastic shifts in LSPR frequencies. One way to induce plasmonic coupling is to use a substrate to bring nanoparticles into closer proximity. A common procedure to implement this analyte-induced plasmonic coupling is to put the analyte of interest onto the surface of a nanoparticle, which then binds a fixed nanoparticle array, producing nanoparticle coupling commensurate with analyte binding. One study by Sharpe *et al.* coupled nanoparticle-bound analyte to a gold nanohole array [41]. This scheme was developed by functionalizing the surface of the nanohole arrays through a novel thiol cortisol derivative to detect a primary antibody followed by binding a secondary antibody functionalized with a gold nanoparticle. This new linker showed high affinity for its antibodies, with low non-specific binding interactions. In another study, silver nanorice were coupled with triangular gold nanoparticle arrays for attomolar detection of hepatitis B virus DNA [42]. A last example, recently carried out in the Van Duyne group, used silver nanotriangle arrays fabricated by nanosphere lithography. After binding an antigen to the antibody-conjugated silver nanotriangles, a secondary antibody attached to a gold nanoparticle was added. The resulting plasmonic coupling between the silver nanotriangles and the gold colloids reduced the limit of detection by three orders of magnitude [43].

Alternatively, it is possible to induce large-scale nanoparticle aggregation in solution through analyte binding. Some studies have applied the aggregation of gold nanorods to detect glutathione and cysteine with high selectivity based on end-to-end self-assembly of the nanorods. The zwitterionic properties of the amino acids, and the affinity of the thiol moiety for the gold surface produced the ordered nanorod arrays [44]. Gold nanorods have also been functionalized with anti-h-IgG, which upon addition of h-IgG aggregate in solution to produce a simple colorimetric assay with a limit of detection of 60 ng/mL [45]. Using a similar scheme, Kotov and co-workers used gold nanorods for highly sensitive and selective detection of 5 pg/mL of the environmental toxin microcystin-LR, which can cause rapid liver failure and liver cancer [46].

**Figure 2:**
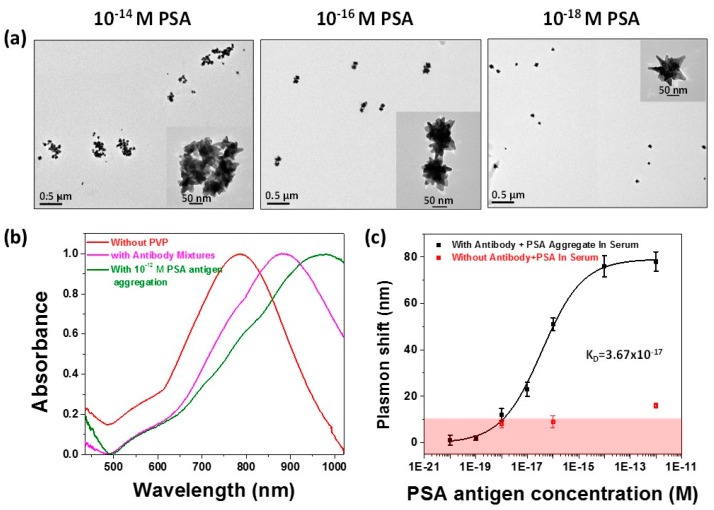
Biosensing using an aggregation assay with PSA protein in serum. Transmission electron microscopy data show a systematic trend of increasing nanostar aggregation with increasing concentrations of PSA, which gives rise to large shifts in the LSPR frequency, (**a**). Raw UV-Vis spectra of the treated nanostars, mixture of antibody-coated nanostars without PSA, and a saturating concentration of PSA revealing shifts as large as 180 nm, (**b**). The binding curve of PSA induced aggregation of antibody-coated nanostars, (**c**) The pink region depicts non-specific binding measured by mixing different concentrations of PSA with nanostars containing no antibody. The binding constant obtained from fitting the data to a single-site model indicates extremely tight binding and a limit of detection of 10^−18^ M PSA [47].

A recent study carried out by Jana *et al.* demonstrated that by removing the polymer-capping agent, polyvinylpyrrolidone (PVP), from the surface of gold nanostars greatly increases the sensitivity of the LSPR frequency to refractive index changes [47]. Additionally, the surfaces of the gold nanostars were functionalized with antibodies for prostate-specific antigen (PSA) and used for ultrasensitive PSA detection. By functionalizing two different batches of PVP-free gold nanostars with PSA antibodies binding to different regions of the protein, a sandwich assay was developed, linking nanostars together through addition of the PSA antigen, see Figure 2. This sandwich assay showed a remarkable limit of detection of 10 attomolar in serum. This low limit of detection was attributed to the increased surface area of the nanostars compared to colloids, enabling more PSA to coat the surface and, hence, more multivalent binding interactions to occur when the nanostars are brought into close proximity.

### 2.3. Biomolecular Conformationally-Gated Amplification

Many efforts have utilized what biology has created to its advantage for signal amplification through the integration of nanoparticles into various biological species. Many efforts have focused on incorporating DNA with nanoparticles for the sensitive detection of DNA through sequence-specific hybridization [48,49,50,51]. Since then, work has been done to improve the detection limits of DNA-nanoparticle assays. One study involved screening endonuclease activity in the presence of inhibiters. Gold nanoparticles are functionalized with single stranded DNA and tethered. Upon addition of deoxyribonuclease (DNAse I), which cleaves the single stranded DNA tethering the nanoparticles together, a colorimetric shift from purple to red is produced. By introducing weak and strong inhibitors to the system, the activity of the enzyme and, therefore, the colorimetric observation of DNAse activity was changed [52]. Others have linked nanoparticles with DNA to detect DNA binding proteins, which upon binding cause a change in conformation of the DNA and hence a change in plasmonic coupling of bound nanoparticles. Li *et al.* recently used these nanoparticle-DNA constructs to detect restriction endonucleases at the femtomolar level [53].

Although the detection of DNA and DNA-binding proteins is extremely useful, extending this concept to proteins would greatly increase the utility and versatility of these assays, since proteins are capable of binding a wider range of substrates. Understanding and detecting proteins and their substrates also has the potential to lead to new drug-based therapies. Nucleases, restriction enzymes, and proteases have been specifically tethered to gold nanoparticles for signal amplification [54]. Protease detection, using a peptide-nanoparticle construct, was developed initially by Guarise *et al.* in 2006 by functionalizing gold nanoparticles with peptide sequences specific for a protease, an enzyme that cleaves proteins. In the absence of a protease, the nanoparticles remain solubilized in solution since the peptide acts as a “capping agent”. However, in the presence of protease, the peptide is removed which induces nanoparticle aggregation and a change in the color of solution from bright red to dark purple [55]. Another study by Hall *et al.* directly detected the conformational change of calmodulin protein attached to silver nanoprisms. Upon binding calcium, calmodulin changes conformation, which in turn changes the LSPR frequency of the silver nanotriangles. This calcium-mediated plasmonic switch showed showed marked changes in LSPR frequency to the addition of a small molecule analyte, which is typically difficult to detect through refractive index-based plasmonic sensing. In addition, real time measurements revealed a rapid, reversible response [56].

Amplification strategies have proven effective at producing drastic reductions in limit of detection, particularly for plasmonic substrates. The implementation of many amplification strategies can be credited to the facile functionalization of gold and silver nanoparticles. However, many of these amplification strategies use delicate, temperature-sensitive enzymes, which would not be amenable to portable, point-of-care devices. Thus, obtaining a low limit of detection with devices that can be implemented easily in the field is still a major challenge in plasmonic sensing.

## 3. The Challenge of Improving Selectivity in Complex Solution

LSPR sensors have had excellent success when implemented without interfering agents yielding high selectivity, however, when introduced to complex solutions, such as blood or urine, there are challenges that must be addressed for accurate and sensitive measurements. In complex biological solutions, many compounds are present that can also bind to the nanoparticle surface, producing false, sizable red-shifts in the LSPR frequency in addition to blocking binding of the analyte of interest. Therefore, in order to implement LSPR sensors for many biological applications, it is imperative that the selectivity of these sensors be improved. Herein, we discuss recent advances in LSPR sensors to improve the selectivity in order to overcome roadblocks caused by complex solutions. The first section below discusses efforts to improve the functionalization layers (or self-assembled monolayers) on the nanoparticle surface for improved selectivity. The next section reviews recent work, which combines the use of biological scaffolds and plasmonic coupling for greater, biologically-mediated selectivity. The final section will examine some recent efforts in which polymers and/or small molecules have created size-selectivity at the nanoparticle surface, providing an additional layer of selectivity for analyte binding.

### 3.1. Improving Selectivity through Functionalization Layers

Typically, in LSPR-based biosensing the selectivity is exclusively determined from the functionalization layer on the nanoparticle surface. Commonly, the functionalization layers are made up of various small molecules, which attract a specific protein of interest to bind. One example is the use of boronic acid ligands for the detection of glucose [57]. Another strategy is to create a self-assembled monolayer of alkanethiols containing carboxylic acids or amine groups, which are amenable to peptide bond formation with proteins such as antibodies. This method can often be highly specific towards the binding of a given antigen. Finally, for DNA and RNA detection it is possible to use DNA-ligated nanoparticles, which hybridize with specific DNA and RNA sequences. This approach has been quite useful for the sensitive and selective detection of specific target sequences, many times associated with diseased states [58,59].

A recently developed methodology to address the problem of selectivity in LSPR biosensing is to include a functionalization layer composed of DNA aptamers. By using a layer of aptamers, which are much smaller than antibodies, the LSPR shift is maximized as the target substrate binds much closer to the nanoparticle surface. In addition, the sensor surface can be regenerated since the layer of functionalization is distinct from the protein being detected. One example is a recent study in which nanomolar levels of protein have been detected and screened by functionalizing gold nanorods with DNA aptamers that are specific for certain proteins of interest [60]. In order to regenerate the sensor surface after the nanorods were functionalized, proteases were added to the system to degrade the bound protein, but did not affect the DNA apatamers bound to the nanoparticle. If antibodies were used instead on the nanoparticle surface they would also be susceptible to protease attack and the sensor surface would not be amenable for reuse. Other areas of interest of that have been investigated by aptamer-nanoparticle technology include small molecule detection [61,62], cancer cell detection [63,64,65], drug molecules [66,67,68,69], DNA [58], proteins, [58,70], and small ions [58]. Although aptamers have comparable affinities for their targets to antibodies and are more robust, limitations to their use come from the rate at which they are discovered along with the cost associated with the development of a novel aptamer.

In addition to attracting a molecule of interest to bind, it is often necessary to repel nonspecific binding using the same functionalized self-assembled monolayers. Thus, the self-assembled monolayer residing on the nanoparticle surface often contains a mixture of molecules aimed at both attracting analytes of interest and repelling unwanted species. Molecules designed to inhibit the binding of unwanted species, or biofouling, is an active and growing area of research, please see reviews on the subject for a more in depth discussion [71,72]. Perhaps the oldest and most widely used anti-biofouling agents are self-assembled molecules composed of polyethylene glycol and oligo ethylene glycol [73,74,75]. These groups have shown impressive resistance to protein adsorption, which has been proposed to be due to the large excluded volume and configurational entropy of the polymer [76,77]. In addition to ethylene glycol, self-assembled monolayers composed of terminal sulfoxide groups have also been shown to be particularly effective at repelling nonspecific binding and exhibit increased solubility in aqueous solutions [78]. More recently, several other types of anti-fouling molecules have been developed, such as saccharide-based molecules [79,80] glycerol-containing moieties [81,82] and peptide-based molecules [83,84,85]. Lastly, an interesting class of zwitterionic anti-fouling agents have been developed, which utilizes common head groups of biological lipids, such as phosphorylcholine [86,87] or 1:1 mixtures of molecules containing opposite charge groups [88]. Similar to agents designed to bind specific molecules of interest in solution, the development of molecules designed to repel unwanted, fouling species is a field in which tremendous progress has been made in the last ten years and is expected to be used in a number applications ranging from biosensing to biomedical engineering.

### 3.2. Improving Selectivity through Biological Scaffolds

Since LSPR-based biosensing does not reveal the molecular identity of the species binding, the detection of unwanted molecules is often problematic. Non-specific binding of biomolecules, or biofouling of plasmonic materials typically results in a “red” shift of the LSPR wavelength, since these species generally increase the refractive index at the nanoparticle surface. On the other hand, biological species, such as proteins, have evolved to bind only one molecule in their active site and carry out specific reactions in the presence of many other similar species. In addition, many biological species can react with a particular analyte and change conformation, thereby changing the spacing of attached nanoparticles. Thus, the selectivity is driven entirely by the biological scaffold, and bound nanoparticles are used as a means of simple colorimetric detection. Since detection stems from nanoparticle coupling (or the spacing between nanoparticles), the changes in LSPR frequency tend to be substantially larger than that observed for refractive index sensing. Therefore, if the nanoparticles bound within the biological scaffold experience biofouling, these red-shifts only minimally effect the measurement. Moreover, many changes in biological conformation can result in nanoparticles moving further apart upon binding a specific substrate, which would cause a blue-shift in the LSPR spectrum, opposite of what is observed for biofouling. This can allow for greater distinction between the binding of unwanted species and the analyte of interest.

**Figure 3:**
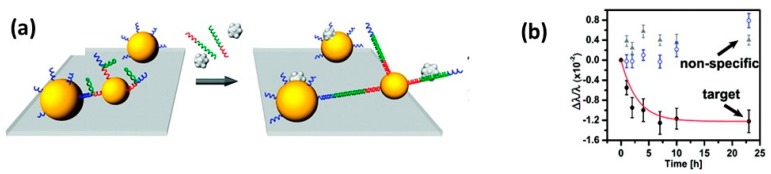
The detection of DNA is demonstrated in complex serum by tethering two gold nanoparticles together through a single strand of DNA with a hairpin loop. In the presence of target DNA, hybridization occurs at the hairpin loop increasing the space between the dimers resulting in a spectral blue shift as a result of decreased plasmonic coupling with great specificity. Reproduced with permission from [91].

The development of the “plasmon ruler” by the Alivisatos group has been an important step towards realizing the goal of using biological scaffolds for greater selectivity. The plasmon ruler is often composed two nanoparticles tethered together by double-stranded DNA [89,90]. Therefore, as an analyte specific for the DNA sequence used to tether the nanoparticles binds and changes the conformation of DNA, an increase in the spacing of the attached nanoparticles produces a blue shift in LSPR frequency from a decrease in plasmonic coupling. This approach was carried out both with purified DNA [90] and in complex biological solutions [91]. The plasmon ruler was also applied to DNA hybridization assays. Since single stranded DNA is much more flexible than double stranded DNA, upon DNA hybridization a rigid DNA dimer is produced increasing the space between the two nanoparticles, decreasing the plasmonic coupling causing a spectral blue shift that is specific for a complementary strand of DNA. Ginger and co-workers were able to detect DNA hybridization in up to 50% serum before nonspecific binding overcame the spectral blue shifts produced by reduced plasmonic coupling, and instead began to produce spectral red shifts, see Figure 3 [91].

Nanoparticle-DNA dimers were also used to selectively detect protein in complex biological solution. In this case, nanoparticles were tethered together using a specific DNA sequence, which produced a hairpin loop. Once the protein bound the hairpin loop, the loop opened up increasing the space between the nanoparticle dimers causing a spectral blue shift [92]. Protein was detected in 20% serum before spectral red shifts occurred from nonspecific binding. Another application used plasmon ruler DNA dimers to study the mechanism of enzyme cleavage of double stranded DNA by the nuclease EcoRV. Upon EcoRV binding to the DNA dimer, the nuclease binds, bends, and cleaves the DNA, which leads to observable changes in nanoparticle dimer spacing for all of these events. In this study, not only was it possible to distinguish nuclease binding, bending and cleavage, but the measurements were carried out in live cells [93]. More complex plasmon rulers that incorporate multiple small nanoparticles tethered to one larger nanoparticle using DNA have also been developed. The DNA sequence used to tether this nanoparticle assembly was specific for caspase-3 cleavage, and these constructs were used to monitor caspase-3 activity in live cells [94]. Plasmon rulers are still an active area of research and it is expected in the future that constructs can be developed exhibiting even greater sensitivity and selectivity. Recently, a three-dimensional plasmon ruler has been developed which shows remarkable sensitivity not just to the distance between nanoparticles, but also the arrangement of the nanoparticles in space, such as dihedral angles [95]. This type of plasmon ruler may pave the way for a new generation of plasmonic biosensors that are exquisitely sensitive to small changes in their environment.

### 3.3. Increasing Selectivity through Size-Selective Films or Shape Complementarity

An approach to increase the selectivity of plasmonic biosensors in complex biological solutions is to incorporate size selectivity into the self-assembled monolayer on the nanoparticle surface. One approach is to use different sized ligands on the nanoparticle surface, which creates pores within the self-assembled monolayer with which only certain molecules can pass [96]. Other groups have also had interest in size selecting proteins using gold nanoislands functionalized with two ligands, a short chain with a reactive group and a long unreactive chain in order to distinguish the protein superoxide dismutase by size from its smaller native state to its large aggregated disease state [97]. The native state protein is able to interact with the reactive short chain due to its small size, while the aggregated protein is too large to enter and does not react. Recently, porous materials such as metal organic frameworks (MOF) or porous silica have been incorporated on the surface of nanoparticle arrays to act as a molecular sieve [98,99,100]. Using a MOF system over silver nanotriangles, selective, bulk refractive index gas detection of CO_2_ and SF_6_ was confirmed through changes in LSPR peak frequency [98].

**Figure 4:**
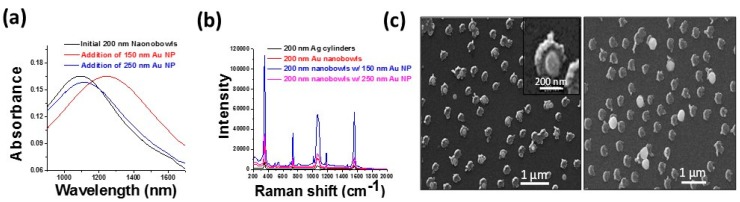
Size-selective sensing of colloidal nanoparticles with 200 nm Au-Ag nanobowl arrays. For nanoparticles small enough to fit into the nanobowls, a large increase in LSPR shift, (**a**) and SERS intensity; (**b**) is observed; (**c**) Scanning electron microscopy images showing the smaller nanoparticles often reside inside the nanobowls, whereas the nanoparticles too large to fit in the nanobowls reside either on top or alongside (unpublished results) [103].

Although nanoporous films and polymers have proven effective for the selective detection of small molecules and proteins, their effect is limited with larger species such as viruses and bacterial cells. Towards the size-selective detection of larger species, a recent study in the Sagle group has taken a different approach to size-selectivity. Instead of incorporating size-selectivity into the self-assembled monolayer on the nanoparticle surface, the shape of the nanoparticle itself was used to select a species of a given size. In this work, gold-silver nanobowls of tunable size were fabricated through the galvanic ion replacement [101] of a silver nanoparticle array made using hole-mask colloidal lithography [102]. A proof-of-concept experiment was carried out by binding gold colloids of a given size to the resulting nanobowl arrays. It was found that when the gold colloids were small enough to enter the interior of the nanobowls, increased LSPR frequency shifts and SERS signal resulted, see Figure 4. These size-selective nanobowls were then used to detect the 95 nm H1N1 virus.

Although great strides have been made towards the development of self-assembled monolayers that can effectively repel unwanted species, the challenge still lies in our ability to simultaneously limit biofouling and at the same time attract small numbers of desired species to bind to the surface. Biological molecules are indeed capable of a high degree of selectivity, performing discerning chemistry in complex solutions regularly. However, the high cost and low stability of these species may challenge their use in portable biosensing devices. A promising approach is to combine shape complementarity directly with the nanoparticle sensing device, and recent advances in our ability to make plasmonic particles of complex shapes should greatly aid in this respect.

## 4. The Challenge of Detecting of Membrane-Associated Species

As evidenced above, developments in nanofabrication and chemistry at the nanoscale have facilitated improvements in limit of detection and selectivity of LSPR based sensing schemes. A rapidly expanding field that is expected to find continued support from LSPR based methods is that of membrane protein and protein receptor sensing. Biorecognition and protein-protein interaction sensing has been a primary application of LSPR since its inception [17,104]. There have, however, been few extensions of this technique to membrane protein interactions and protein receptors. The drive to develop new methods to characterize and screen, especially in a high-throughput manner, membrane protein systems comes from rapid advances that are being made in understanding protein receptors. In particular, G protein-coupled receptors have emerged as predominant targets for pharmaceutical therapies [105,106]. Assays that target these species often involve labels or indirect measurements that may influence a protein system or have limited throughput [107]. It is, thus, desirable to develop sensitive, label-free methods to characterize these species. It is important that new methods are easily scalable to address throughput problems, and that the surrounding sensing environment reflect that of the native protein. Lipid bilayers formed on hydrophilic supports (supported lipid bilayers) are a well-characterized model membrane system that can be prepared to mimic different cellular membrane environments [108]. While still challenging to engineer metal nanoparticle systems for refractive index based sensing of such systems, there are now several examples of LSPR based sensors that prepare supported lipid membranes in close proximity to metal nanoparticles. The primary contributions to this new field will be reviewed, as well as challenges to be addressed.

### 4.1. Supported Lipid Membranes

Membrane-associated biological species are often studied through the use of detergents, surfactants and unilamellar lipid vesicles. SPR and quartz crystal microbalance (QCM) have been used extensively to study biomolecular-membrane interactions and have been reviewed elsewhere [109,110]. The electromagnetic field decay lengths for LSPR substrates are considerably smaller than that for SPR, reducing the effective sensing volume. Thus, with the exception of a few liposome-based studies [111,112], LSPR has been predominantly used with the supported lipid bilayer systems, in which the lipids, and associated species, reside in close proximity to the support.

Formation of supported lipid bilayers is often carried out on supports, such as glass, quartz, or mica [113,114,115]. Vesicle fusion is the most common mechanism of supported lipid bilayer formation on these hydrophilic surfaces. Vesicle fusion occurs when vesicles in solution adsorb to the surface and rupture leading to bilayer formation across the entirety of the hydrophilic support [108]. The presence of a hydration layer, approximately 1 nm thick, between the support and the inner lipid leaflet allows the bilayer to remain laterally fluid and enables diffusion of lipids, small molecules and proteins to occur in the plane of the membrane. This allows supported lipid bilayers to mimic the fluidity of a natural membrane. Diffusion coefficients of fluorescently labeled lipids determined through fluorescence recovery after photobleaching (FRAP) are approximately 1 μm^2^/s on glass [108].

In addition to hydrophilic surfaces, preparation of supported lipid bilayers on materials such as aluminum oxide, titanium oxide, and gold have also been described [116,117]. In these systems bilayer formation is often not spontaneous. Furthermore, diffusion coefficients and mobile fractions of lipids in these bilayers are often diminished, suggesting larger interactions between the surface and the bilayer. In general, limiting interactions between the support and any protein, specifically transmembrane species, is ideal. Interactions with the support may denature the protein or induce structural changes that may ultimately influence binding or activity [118]. It is interesting to note that developments in preparing lipid bilayers on different surfaces, such as aluminum oxide, titanium oxide, or gold, may expand the applications of lipid bilayers to new functional materials, such as novel biosensors or biologically inert surfaces [119].

### 4.2. LSPR Based Membrane Biosensors Using Supported Lipid Bilayers

**Figure 5:**
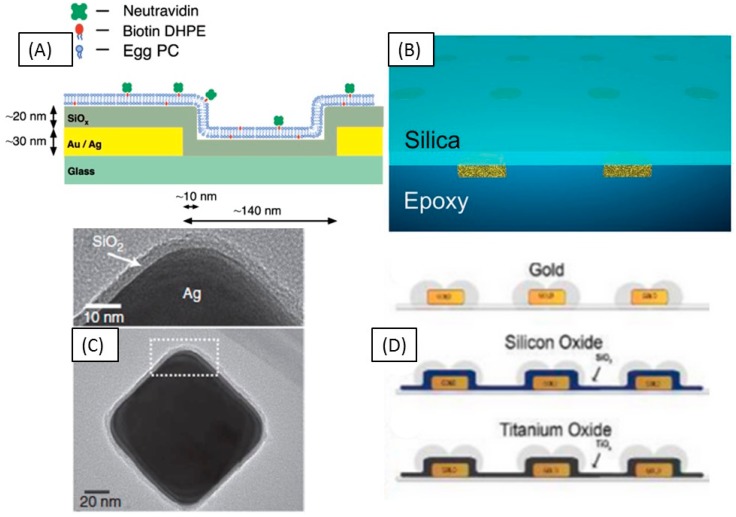
Current bilayer LSPR based sensing schemes employing silica coatings. (**a**) Nanohole arrays coated with about 20 nm of silica; (**b**) Nanodisks embedded in an optical epoxy coated with about 10 nm of silica; (**c**) Ag nanocubes coated with thin layer of silica; (**d**) Protruding nanodisk arrays coated with about 10 nm of silicon oxide or titanium oxide [117,120,121,122].

The first example of a sensor diverging from the SPR concept was constructed with nanometric holes in a thin gold film [120]. Thin metal films perforated with nanoholes exhibit LSPR modes that are confined to the nanohole (Figure 5). These structures were prepared over quartz surfaces and formation of a supported lipid bilayer within the nanohole was demonstrated. Furthermore, both formation of the supported bilayer and binding of neutravidin to biotinylated lipids could be monitored in real time, allowing accurate determination of kinetic parameters. Bulk refractive index sensitivity measurements of the substrate were 180 nm/RIU. Currently, a common approach to make a surface compatible for lipid adsorption has become to encase the structure or surface in silica. Following the first membrane LSPR concept, these same nanohole structures were encased in a thin film, approximately 20 nm, of silica [121], which eliminates quenching, while simultaneously creating a surface that is amenable to fluid bilayer formation. Fluorescence recovery after photobleaching measurements (FRAP) is then an easy confirmation of fluid bilayer formation. As was found with the silica over nanohole configuration there is a concomitant drop in refractive index sensitivity upon coating with silica. Bulk refractive index sensitivities for these structures may be found in Table 1.

One of the most recent supports aimed at lipid membrane based sensing has prepared flat surfaces with embedded nanoparticles. In this method nanodisc arrays are prepared and embedded in an optical epoxy [123]. A technique termed template stripping is then used to remove the epoxy film containing nanoparticles. Atomic layer deposition is used to deposit a thin silica layer on the other flat side of the film that had initially interfaced with a silicon wafer. Through this technique, diffusion coefficients of 2.06 μm^2^/s were found, which are the highest reported for an LSPR based support. Another group has adopted a similar methodology as above, taking advantage of a nanoplasmonic sensing scheme developed in 2010 [124]. Thin films of dielectric material are deposited over nanoparticle arrays prepared by hole-mask colloidal lithography. In this work, the arrays of surface bound nanoparticles were covered with a thin film, about 10 nm, of either silicon oxide or titanium oxide and vesicles were adsorbed to the surface [117]. Bulk refractive index sensitivities for the bare gold array and for the silica-coated structures were found to be 232 and 110 nm/RIU, respectively. The kinetics of vesicle adsorption and bilayer formation were assessed by LSPR shift. Spontaneous vesicle rupture and bilayer formation were found to occur on the silica-coated substrate while intact vesicles were found to remain on the bare gold and titanium oxide coated arrays.

**Table 1:** Sensitivities of LSPR based bilayer constructs after being coated with a dielectric layer.

Sensor Substrate	Bulk Sensitivity (nm/RIU)	Coating Thickness (nm)	Reference
Au nanoholes	113	20	[120]
Ag nanoholes	75	20	[120]
Flat Au nanodisks	[4.5 nm/(nm of Al_2_O_3_)] 150 (approximate)	10	[122]
Ag nanocubes	123	3.9	[121]
Protruding Au nanodisks	110	10	[116]

Colloidal nanoparticles deposited on a surface and dispersed in solution have also been used to measure lipid adsorption and protein receptor binding. One of the first experiments implemented a dark field microscopy configuration to measure lipid bilayer formation over single gold nanorods [125]. Silver nanocubes in solution have also been demonstrated to be efficient LSPR-based lipid membrane supports [122]. Silver nanocubes prepared through the polyol method were coated with a thin, 4 nm silica shell, through the Stober process. The quadrupolar mode of the Ag nanocube was used for measurements and bulk refractive index sensitivities were similar to the nanohole arrays. In this system, the adsorbed lipids and protein species are significantly closer to the metal nanoparticle, enabling more proteins to exist within the decay length of the sensor. As few as 800 streptavidin molecules were found to adsorb to a single nanocube, resulting in an LSPR shift as large as 6 nm. After calibrating their assay the authors applied the nanocube LSPR sensor to quantify the micromolar binding affinity (K_d_) of an incompletely characterized protein kinase scaffolding protein, Ste5 [126]. One of the primary advantages of this solution phase system over surface bound systems is an ability to increase signal by increasing particle concentration.

Through all of the LSPR based membrane sensors developed to date, there have been attempts to develop indirect sensing schemes by displacing the membrane from the surface of the nanoparticle transducer. The smallest distance has been that of the silver nanocube and the largest are 10–20 nm, which effectively represent the extent of the plasmon field in many LSPR systems. Additionally, as can be seen from Table 1, the sensitivities from the substrates fabricated have all clustered around similar values, which represent current limitations for the LSPR substrates. Methods for coating complex geometrical structures that may have higher sensitivities or larger decay lengths may be advantageous for increasing refractive index sensitivity and applicability of these substrates for different lipid or protein systems [104]. Indeed, there are few examples of the direct application of LSPR based membrane sensors currently, the majority of which have been carried out by Oh and colleagues [122,127,128,129]. Sensitivity issues may be one of the principle components of this. In situations where equipment limitations inhibit large signal to-noise ratios or the molecular species being used result in small refractive index changes, such as the case of small molecule ligands binding lipophilic proteins, increases in refractive index sensitivity are essential.

Finally, as will be addressed below, the combination of nanofabrication with microfluidics will allow for increased scalability and ultimately the potential for multiplexing capabilities. Miniaturization is one of the large benefits of nanoparticle systems over analogous methods such as SPR. It is expected that the coordinated development of these fields will yield powerful, high-throughput, array-based platforms for biosensing and ligand screening.

## 5. The Challenge of Incorporating LSPR Biosensing into Point-of-Care Diagnostic Devices

LSPR-based biosensing provides sensitive, label-free, facile detection with devices that are relatively inexpensive to fabricate. Thus, these biosensors are ideal for resource limit environments, where cost, rapid detection, and transportability are paramount [130]. This section will first discuss a few LSPR platforms that are particularly amenable to portable point-of-care diagnostic applications. In order to fully realize the potential of this technology to resource-limited environments, incorporation into multiplexed, microfluidic devices is necessary. This will allow for more accurate diagnostic measurements since it has been established that multiple biomarker assays generally lead to less false diagnosis than single biomarker tests. The last few sections will discuss efforts made in recent years towards multiplexed LSPR biosensing measurements and the incorporation of plasmonic sensors into microfluidic platforms.

### 5.1. Plasmonic Point-of-Care Diagnostics

**Figure 6:**
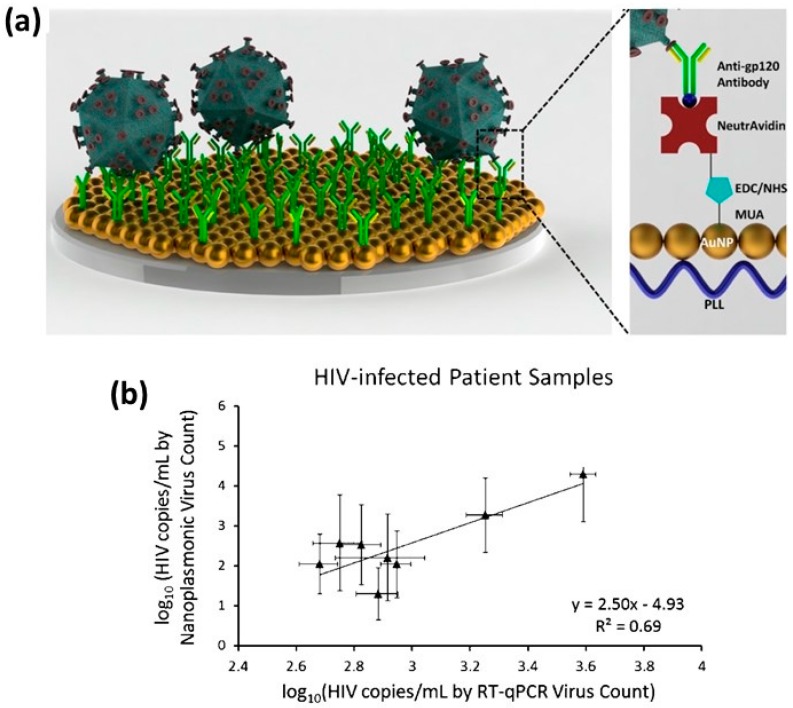
(**a**) An HIV detection assay is depicted which relies on carbodiimide chemistry to cover the surface with antibodies specific for HIV subtypes; (**b**) The plasmonic system can detect and distinguish between HIV subtypes A, B, C, D, E, G in patients with HIV [136].

An initial application of LSPR biosensing platforms for clinical use was carried out by Haes *et al.* in 2005 and detected a biomarker, amyloid-derived diffusible ligand (ADDL), for Alzheimer’s disease [131]. An array of 90 nm silver nanoprisms was fabricated using nanosphere lithography [25], which were functionalized with anti-ADDLs and exposed to varying concentrations of ADDLs. After this, additional anti-ADDLs were added to boost the detected signal. This was then successfully tested in cerebrospinal fluid from Alzheimers patients. Since then, many plasmonic sensors have been developed for potential POC use for various diseases. One disease that disproportionately affects developing countries is HIV, thus many LSPR platforms have been developed towards the detection of the HIV virus or HIV-associated proteins and DNA [132,133,134,135,136]. A recent study by Demirci *et al.* accurately captured, detected, and quantified different subtypes (A, B, C, D, E, and G subtype panel) of HIV with accuracy of 98 ± 39 copies/mL for Virus subtype D. Tests were conducted in whole blood samples from HIV patients on a gold platform using immunochemistry to trap the virus particles monitoring wavelength shifts with high reproducibility, sensitivity and specificity, see Figure 6 [136]. In addition to HIV, other point-of-care plasmonic detection schemes have been developed to detect viruses [137,138,139,140] and cancer [141,142,143] in resource limited environments.

**Figure 7:**
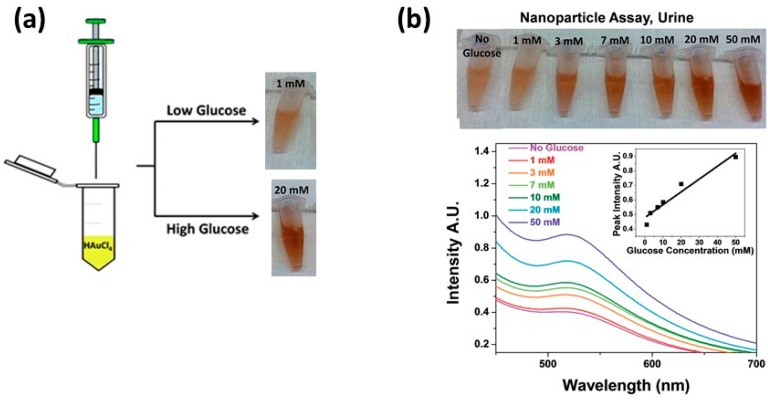
A schematic for glucose detection through nanoparticle formation, (**a**) that was tested in whole urine samples spiked with glucose; (**b**) As the samples were heated under alkaline conditions, there was a colorimetric increase in intensity with increasing concentrations of glucose [151].

Another disease that has a large effect on the livelihood of people in the developing world is diabetes [144]. Unfortunately, current electrochemically-based glucose sensors require batteries which limit their use and are often prohibitively expensive. Therefore, many efforts toward developing novel biosensing devices that do not rely on the current electrochemical method have been developed, including LSPR technologies [145]. Rahakumary *et al.* successfully demonstrated the detection of glucose in urine by functionalizing thiol capped gold nanoparticles with the enzyme glucose oxidase, while in the presence of glucose aggregated causing a color change from red to purple with a limit of detection of 100 μg/mL [146]. Several studies have successfully used localized surface plasmon resonance platforms to detect glucose in biological fluids such as urine [147], blood plasma [148], serum [149] and cerebrospinal fluid [150]. Efforts have been made to simply and rapidly detect glucose in biological samples for point of care diagnosis and treatment. One recent example is a study by Unser *et al.*, who developed a simple, enzyme-free colorimetric sensor for glucose based on the ability of glucose to form gold nanoparticles in solution under basic conditions. Further tests detected glucose in 20% mouse serum yielding color changes similar to the *in vitro* assay. In addition, the assay was shown to be effective at detecting glucose in whole urine samples colorimetrically. As shown in Figure 7, urine samples containing significant amounts of glucose appear darker in color than those with less glucose, and a linear response was observed for a wide range of glucose concentrations from 1.25 mM to 50 mM [151].

### 5.2. Multiplexed LSPR Platforms

Another component to being able to carry out point-of-care diagnostics, particularly in resource-limited environments, is to incorporate these simple optical devices into portable, multiplexed and microfluidic platforms. This technology will allow for rapid, portable, inexpensive detection with significantly smaller amounts of biological fluid. Multiplexed measurements also have the advantage of producing more accurate, systematic measurements, since all the samples can be measured at the same time, with the same reference solutions. This section will first discuss recent advances in LSPR-based multiplexed biosensing devices, which range from spot-plate assays to single nanoparticle devices. Lastly, advanced devices incorporating nanoparticle arrays into microfluidics will be reviewed.

#### 5.2.1. Multiplexed Plasmonic Arrays

Multiplexed LSPR biosensing in solution has been carried out with bulk solutions of nanorods, which display unique spectral features based on aspect ratio of the nanorods themselves [152]. By functionalizing nanorods of different aspect ratio with different antibodies (human IgG, rabbit IgG and mouse IgG, respectively), the LSPR change associated with individual IgI binding can be easily distinguished. However, nanoparticle array-based LSPR substrates can often provide a higher degree of uniformity and reproducibility. To realize multiple measurements on one LSPR solid substrate Endo *et al.* [153] made a plasmonic chip by making core-shell structures on the surface. Silica nanoparticles were self-assembled to form a monolayer on the surface of gold-coated substrates, and another layer of gold was deposited on top, forming a shell. Multiple spots were created by packing the spheres so that the separation between adjacent structures was approximately 1 mm. Thus, high-throughput measurements of different antibody-antigen pairs were accomplished on a single chip.

**Figure 8:**
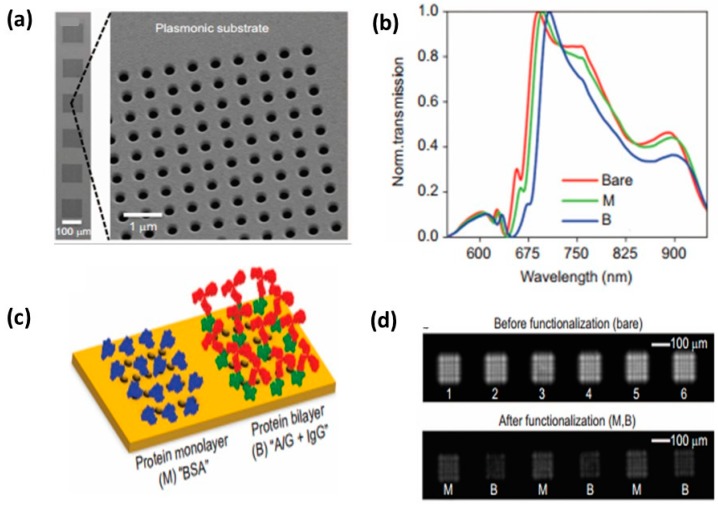
Nanohole substrate for multiplexed biosensing and lens-free imaging. (**a**) SEM images of 6 pixels of nanohole arrays. Each pixel is 100 µm × 100 µm with hole size 200 nm; (**b**) Schematic of nanohole arrays coated with different proteins: Monolayer of BSA (M), bilayer of protein and IgG (B); (**c**) Transmission of bare nanohole arrays (red), with protein monolayer (green) and bilayer (blue); (**d**) diffraction patterns of nanohole arrays before and after functionalization with protein [156].

However, transparent arrays, which change color upon interacting with a given biological species, are often desirable, since standard UV-Vis instrumentation is generally carried out in transmission mode. Two protocols for making transparent multiplexed nanoparticle arrays, which could ideally interface with standard instrumentation, have been developed. The first protocol used “gold staples” in which vertical gold bars are evaporated onto a substrate leaving a small space in between. Upon annealing followed by a second gold deposition, a shadowing effect is created in the middle of the “gold staples”, creating a gradient of different gold thicknesses where the gold meets the glass substrate. This gradient of gold thickness in turn created a gradient of plasmon resonances, which could be utilized for multiplexed measurements. In addition, to further increase the multiplexed capabilities of the substrate, the thickness of the second gold deposition can be changed to create a gradient in the perpendicular direction [154]. Multiplexed measurements of atrazine binding to anti-IgG atrazine antibody are then studied in these substrates. Another approach to creating multiplexed, transmission-based LSPR substrates is to use colloidal lithography to make nanoparticle arrays in which the final metal deposition step is carried out through a mask. This created defined spots containing uniform nanoparticles on the substrate, which were separated by a distance defined by the mask [155]. Using this method, nine individual spot areas containing nanoparticle arrays with 190 m diameter and 40 nm height are built on the substrates. With an LSPR imaging instrument in which white light is transmitted through the sample, split into component wavelengths using liquid crystal tunable filters, and detected using a CCD camera, data collection occurs simultaneously over the whole chip. Lastly, recent work has been carried out using nanohole arrays towards multiplexed biomolecule screening [128,156]. Cetin *et al.* made a portable chip with 100 μm × 100 μm pixels consisting of plasmonic nanohole arrays, which upon binding a biological analyte changed the amount of transmitted light. These highly portable, lens-free devices utilized a CMOS (Complementary Metal-Oxide-Semiconductor) imager chip with computational reconstruction to record differences in diffraction patterns as protein bound to the plasmonic nanohole arrays illuminated at the LSPR wavelength, see Figure 8. The images can be reconstructed to make a chip of multiple sensors only 10 μm × 10 μm each for further high throughput measurements [156].

#### 5.2.2. Multiplexed Single Nanoparticle LSPR Sensing

Another approach to enabling several LSPR biosensing measurements on a single chip is through single nanoparticle LSPR. Single nanoparticle measurements not only have the advantage of using smaller amounts of sample and nanoparticle material, but are also capable of measuring smaller amounts of bound analyte [157]. Single nanoparticles can be easily distinguished on a chip using dark-field microscopy [158]. Taton *et al.* [159] did some early work in which single nanoparticles were used to detect different DNA sequences. Nanoparticles of different size were functionalized with DNA oligonucleotides. The resulting color changes in the single nanoparticle LSPR spectrum showed high sensitivity and selectivity for DNA in an array format. More recently, biosensing with single nanoparticles of different shape has been extended towards the detection of different cancer cell lines [160]. In addition, Christina *et al.* [60] monitored the response of aptamer-functionalized nanorods of different length to protein binding using dark field microscopy. The different aptamer-protein pairs were measured on the same chip according to the unique spectral signature of nanorods of different length. To further improve the throughput, recent work ofAhijado-Guzmán *et al.* [161] incorporated single gold nanorods into a flow cell. Nanorods were stabilized and functionalized with DNA and Ni^2+^ bound nitrilotriacetic acid (NTA), which was then complexed with different proteins containing his-tags (s1ZipA, s2ZipA or MinC). By sequentially depositing different protein-functionalized nanorods into the flow cell and recording their position by dark-field microscope, multiple measurements of target protein binding could be carried out by measuring different positions along the channel.

### 5.3. Microfluidic LSPR Biosensing Devices

Measuring multiple biomarkers in real time with complex biological solutions would be ideal to effectively carry out point-of-care diagnostics. Although multiplexed assays mentioned above are facile and often inexpensive, a continuous flow format would allow for more rapid measurements with decreased volumes of solution, particularly when more than one step is involved in the sensing scheme under study. Microfluidic platforms, containing channels for continuous flow, provide a means to carry out these highly multiplexed, rapid reactions.

**Figure 9:**
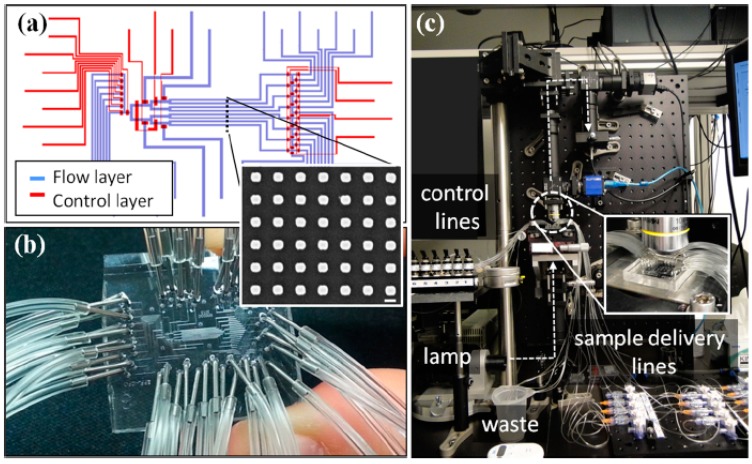
Schematic of the 8-channel microfluidic device containing both control and flow layers, (**a**); Each channel contains multiple “spots” of gold nanodisc arrays for multiplexed biosensing measurements, (**b**) (inset has a scale bar of 200 nm); Overview of the optical setup used to measure the plasmonic response of the nanoparticle spot arrays within the channels, (**c**) Reproduced with permission from [162].

A recent study created a microfluidic device containing nanodisc arrays in eight different continuous flow channels. Electron beam lithography is used to “write” nanodisc spot arrays into the microfluidic channels, which are then sealed with polydimethylsiloxane (PDMS) [162], see Figure 9. Precise flowing and independent control of each channel is possible by interfacing the pumps with a LabView program. Since each channel contains several spots of gold nanodisc arrays, this design can give many sensing sites from only eight channels. Kinetic binding data is then demonstrated with this device for various cancer biomarkers, such as human alpha-feto protein and prostate specific antigen, with a limit of detection of 500 pg/mL in 50% human serum. Another recent study by Lee and co-workers developed a 50-channel microfluidic system using silver nanohole arrays coated in silica, which conveniently seal in a facile manner to PDMS. The capability to measure both binding kinetics and affinities simultaneously was then demonstrated with Cholera Toxin B subunit binding to ganglioside lipids in solid supported lipid bilayers present in the microfluidic channels [163].

In addition to interfacing microfluidic channels with nanoparticle arrays, work has also been carried out towards single nanoparticle sensing in microfluidic channels. Chen *et al.* [164] combined single nanoparticle measurements with microfluidics by flowing a solution containing gold nanorods through microfluidic channels. The PDMS/glass microfluidic channels were functionalized with negatively charged silane, which allowed the positively charged, CTAB-coated gold nanorods to electrostatically adhere to the bottom of the channels. Using dark-field microscopy, the LSPR spectrum of single gold nanorods were measured as various concentrations of cytokine samples in serum were flowed through the channels. This device was able to generate multiple biosensing measurements within a single microfluidic channel, with ultralow sample volumes. This pioneering work paves the way for highly multiplexed, microfluidic devices, which will undoubtedly be useful in a number of biosensing applications.

Although interfacing LSPR biosensing with on-chip technologies is in its infancy, the current success has generated enormous potential. Combining single nanoparticle LSPR with microfluidics, in particular, has the ability to create devices with unprecedented multiplexing capabilities. These devices will allow for the simultaneous measurement of multiple binding partners in complex solutions, within a single microfluidic channel. In addition, improvements in inexpensive, scalable fabrication should also enable the integration of LSPR technology with other biological on-chip devices.

## 6. Prospective and Conclusions

Localized surface plasmon resonance is a leading technique for label-free biosensing, with both facile, colorimetric detection and the ability to interface with portable, multiplexed devices. Although the potential for LSPR technologies to greatly impact biosensing is high, clear challenges and limitations exist. One key challenge is to increase the sensitivity of the devices and improve the limit of detection of desired analyte. The most promising approaches in this direction are amplification strategies, since these many times lead to decreases in limit of detection by several orders of magnitude. A few recent studies highlighted in this review have combined more than one amplification strategy, such as enzymatic amplification and nanoparticle reactivity, to yield remarkably low limits of detection. Another challenge discussed is selectivity in binding the analyte of interest while minimizing biofouling, particularly in complex biological solutions. A large range of advancements in both self-assembled monolayers, which combine attracting the molecule of interest with repelling unwanted species, and the use of biological scaffolds appear promising in this respect. While biological scaffolds seem the more selective route, the use of biological species makes these devices suceptable to denaturation and less portable. An additional strategy is to incorporate shape or size complementarity directly into the nanoparticle substrate shows perhaps the most potential in yielding robust, selective devices. Towards these goals, the tremendous growth in the field, particularly in creating nanoparticles of complex shape and reactivity, should greatly aid in improving limit of detection and overall selectivity.

The last two challenges reviewed involve the construction of devices for drug screening and point-of-care diagnostics. Since more than half of all drug targets are membrane proteins, LSPR technologies could prove extremely useful as an inexpensive, high throughput, label-free option. Unfortunately, solid supported lipid bilayers, the most useful lipid systems for LSPR, only form readily on silica substrates, and not plasmonic substrates. Thus, plasmonic devices for the detection of membrane-associated species often contain a layer of silica on top to interface with the solid supported bilayer, which reduces sensitivity. Although some promising plasmonic devices have been fabricated to interface with solid supported lipid bilayers, facile detection is problematic due to low sensitivity. At the same time, recent interest has grown enormously towards multiplexed and microfluidic LSPR biosensing platforms, leading to advances in fabrication and microscopy. Undoubtedly drug-screening devices will also benefit from these advances. Future point-of-care devices will most likely require interfacing LSPR technologies with cell lysing and separation platforms for true lab-on-a-chip applications.
